# Orographic amplification of El Niño teleconnections on winter precipitation across the Intermountain West of North America

**DOI:** 10.1038/s44221-023-00163-9

**Published:** 2023-12-04

**Authors:** James H. Stagge, Max C. A. Torbenson, Kyungmin Sung, Benjamin Phillips, Daniel G. Kingston

**Affiliations:** 1 Department of Civil, Environmental and Geodetic Engineering, The Ohio State University, Columbus, OH USA; 2 Department of Geography, Johannes Gutenberg University, Mainz, Germany; 3 Department of Food, Agricultural and Biological Engineering, The Ohio State University, Columbus, OH USA; 4 School of Geography, University of Otago, Dunedin, New Zealand

**Keywords:** Atmospheric science, Hydrology, Climate change

## Abstract

A large proportion of western North America experiences regular water stress, compounded by high seasonal and interannual variability. In the Intermountain West region, the El Niño/Southern Oscillation (ENSO) is a critical control on winter precipitation, but the nature of this signal is entangled with a combination of orographic effects and long-term climate trends. This study employs a spatially distributed, nonlinear spline model to isolate ENSO impacts from these other factors using gauge-based observations starting in 1871. In contrast to previous modelling approaches, our approach uses original gauge data, without shortening the record to accommodate a common period. This enables more detailed separation of ENSO effects from the confounding influence of topography and long-term trends, whereas the longer time frame permits more robust correlation with the ENSO signal. Here we show that the complex topography of the Intermountain West exaggerates the underlying ENSO signal, producing a 2.3–5.8 times increase in the range of ENSO-induced precipitation changes along high-elevation western slopes relative to lower elevations. ENSO effects on winter precipitation can be as large as ± 100 mm at high elevations. Further, our approach reveals that the previously recognized dipolar pattern of positive (negative) association of ENSO with precipitation in the south (north) manifests as an incremental relationship in the south but as a near-binary switch in effects between El Niño and La Niña in the north. The location and extent of the strongest precipitation differences vary during the positive and negative ENSO phases within each region. The intricacies of these spatial- and elevation-based modulations of ENSO impacts are especially informative for the northern centre of this dipole, where ENSO-precipitation relationships have previously been difficult to resolve.

## Abstract (web summary)

The El Niño/Southern Oscillation (ENSO) is a critical control on winter precipitation in the Intermountain West of North America but is impacted by mountainous terrain and climate change. This study isolates ENSO impacts from these complicating factors using a spatially distributed, nonlinear spline model applied to long-term gauge observations.

## Main

Much of western North America is classified as under extremely high water stress^1^ due to low annual precipitation (200–500 mm per year), frequent multi-year droughts and a reliance on reservoirs to satisfy agricultural, municipal and ecological demands^2^. In Mexico and the USA, 50.3 million and 50.9 million urban inhabitants face water scarcity for at least one month of the year, respectively, most of whom live in western states^3^. In addition to municipal water risk, nearly all of the region’s agriculture is irrigated due to low growing-season precipitation, further emphasizing the importance of reservoir storage and groundwater extraction^2^. From 1980 to 2022, droughts in the USA accounted for 13.5% of all losses from weather and climate disasters, totaling US$309.4 billion in consumer price index (CPI)-adjusted dollars^4^. The ongoing drought in the western USA represents the driest 22-year period since at least 800 ce (common era)^5^ and is testing the resilience of water management systems. For example, Lake Powell and Lake Mead, the two largest reservoirs in the USA by volume, were at their lowest stage since completion in 2022^6^. The situation was similar in northern Mexico, where the Las Virgenes reservoir reached as low as 17% of capacity, placing it at risk of structural failure, amid a decrease in planted area due to low irrigation storage^7^.

The climate of western North America is characterized by strong seasonality and low precipitation during the growing season^8^. Winter precipitation therefore provides critical water to be stored for seasonal irrigation or used as a multi-year drought buffer^9^. Late summer also provides seasonal precipitation for parts of the southwestern USA and northern Mexico, but this summer precipitation is driven by monsoon patterns unlike those explored here. This study focuses on winter precipitation in the mountainous region between 116° and 99° W, sometimes referred to as the Intermountain West. This region is part of the North American Cordillera, which forms a continental hydrologic divide and headwaters for some of the largest rivers in North America. Mountainous terrain complicates precipitation patterns due to the orographic effect, wherein moist air is forced upwards along windward slopes and condenses, leading to excess precipitation along predominantly windward slopes and decreases along leeward slopes^10^. Motivated by the high stress on the region’s water supply and its reliance on high-elevation winter precipitation, the primary goal of this study was disentangling and understanding how elevation moderates the effects of the El Niño/Southern Oscillation (ENSO)^11,12^ on winter precipitation.

ENSO has an established impact on precipitation across western North America, particularly along the Pacific coast^13–15^. The El Niño phase stems from surface warming in the central and eastern tropical Pacific Ocean, leading to a weakening of low-level surface easterly winds, whereas the La Niña phase produces the opposite pattern^11,12^. During El Niño years, winter precipitation tends to increase in the southwestern USA, northwestern Mexico and the Gulf of Mexico coast due to an extended Pacific jet stream that funnels moisture eastward along the US–Mexico border^16–18^. During these conditions, a northern centre of ENSO influence experiences the opposite, decreased precipitation^13,15,16,19,20^. Because these two centres of ENSO influence are opposed, they are sometimes referred to as a precipitation dipole^16^. We use this dipole terminology throughout the study to refer to these opposing areas of influence on precipitation.

Isolating interannual ENSO effects is further complicated because it is overlaid onto a signal of anthropogenic climate change^21,22^. The southwestern USA and Mexico are consistently identified as hotspots for decreasing precipitation trends during the last century^23,24^ and in future projections^22,25–27^. Situating these drying trends relative to pre-industrial climate has shown that recent decades are uniquely dry among the last millennium^5,28^.

**Extended Data Fig. 1:**
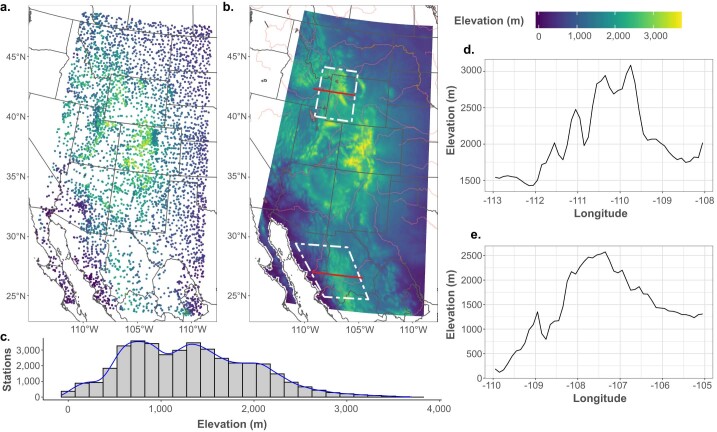
Regional topography. Topography of the study region, shown by (a) all GHCN-D gauges included in the analysis and (b) SRTM elevation data used for predictions. Subfigure (b) shows the location of dipole regions in white, with the East-West transect in red. The overall distribution of station elevations is shown in Subfigure (c). Elevation along the (d) North-Wyoming and (e) South-Sonora were calculated by extracting SRTM elevation data along each transect.

**Extended Data Fig. 2:**
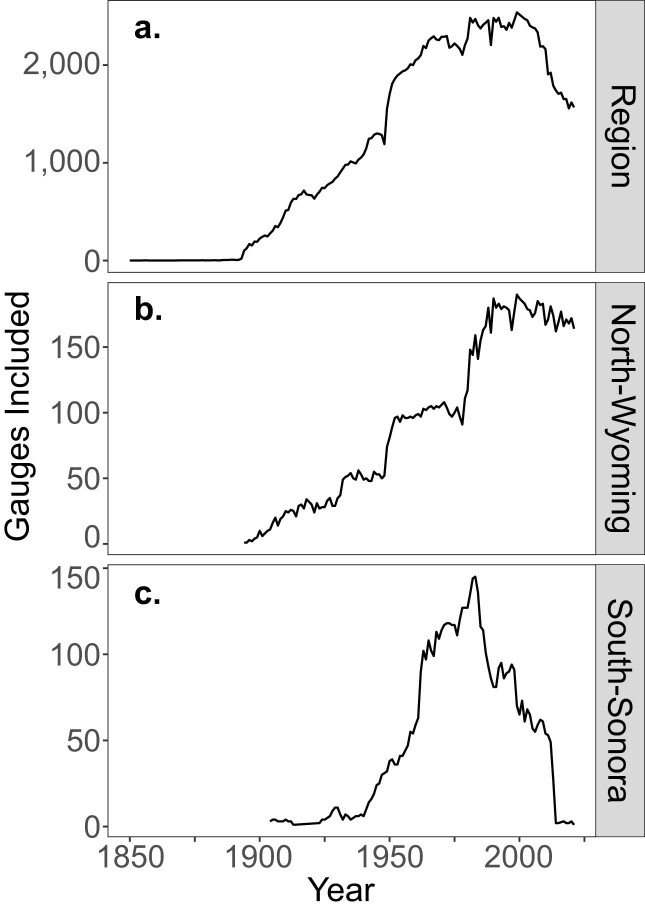
Precipitation gauge availability. Gauge availability by year for (a) the full region, and dipole regions for the (b) North-Wyoming and (c) South-Sonora models.

**Extended Data Fig. 3:**
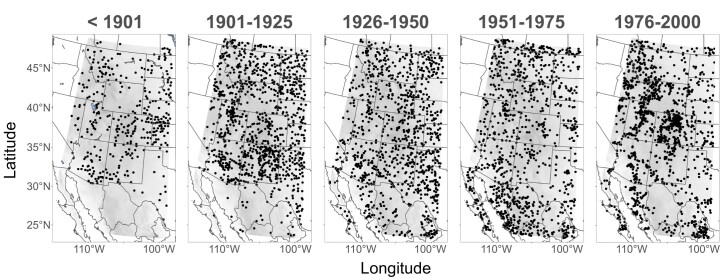
Spatial gauge availability by year. First year of precipitation gauge availability by 25 year segments.

ENSO effects are clearest, and most well studied, along the Pacific coast^16^; however, the blocking effects of the Sierra Nevada mountains^29^, complex topography of the North American Cordillera and pronounced precipitation trends^22,25^ make quantifying the effects of ENSO in the Intermountain West more challenging^19,30^. This study seeks to address this by isolating the effect of ENSO on winter precipitation from its complex interactions with elevation and multi-decadal climate trends. Disaggregation of the ENSO signal was made possible by applying a novel Generalized Additive Model (GAM), wherein each term is modelled spatially using nonlinear spline surfaces. This statistical modelling approach is unique among prior ENSO studies and enables a more detailed separation of ENSO effects from the confounding influence of topography and long-term trends, while also permitting varied record lengths that provide up to 148 years of temporal coverage, longer than previously available. Further, the dense but irregular network of ground-based gauges provides greater understanding of spatial and orographic effects, without the potential smoothing effects of reanalysis models or spatial averaging. The resulting model was applied to 4,287 gauges covering nearly a century and a half of winter precipitation, encompassing the north and south ENSO dipole centres from southern Canada to central Mexico (Extended Data Figs. 1–3). The results show the effects of ENSO and topography, jointly and in isolation, indicating a potential for high resolution ENSO-based forecasts of winter precipitation^13,31^ with usable confidence intervals for water resource management.

## ENSO effects on winter precipitation

To disentangle ENSO effects from other factors, a GAM was developed to explain December–February precipitation, *P*_DJF_, as a function of gauge location, elevation, long-term trends and ENSO:

1 $$\log ({P}_{{{{\rm{DJF}}}}})=f(x,y)+f({{{\rm{elev}}}})+f((x,y),\,{{{\rm{year}}}})+f((x,y),\,{{{\rm{ENSO}}}})$$

Greater statistical detail on GAMs is provided in the Methods. Winter precipitation was modelled using a Tweedie distribution to account for positively skewed distributions, while also accommodating zero precipitation years. The first two terms of this model are fixed temporally, representing location and elevation, respectively. When summed, these terms model climatology as the sum of spatial and orographic effects. The final two terms, *f*((*x*, *y*), year) and *f*((*x*, *y*), ENSO), change through time and respectively represent multi-decadal climate trends and interannual ENSO effects, modelled spatially. In addition to incorporating skew and zero values, the GAM benefits from allowing complex spatial patterns, while explicitly modelling each term in equation (1) to measure their relative effect. Gauge-wise correlation, regional averages or composite analyses used in prior ENSO studies^30,32^ can capture spatial complexity but cannot easily disaggregate elevation or long-term trends, as done here. Two model versions were fit as part of this study, referred to here as the ‘regional’ and ‘dipole’ models, which capture the full Intermountain region and individual north–south subsets of the dipole, respectively.

**Extended Data Fig. 4:**
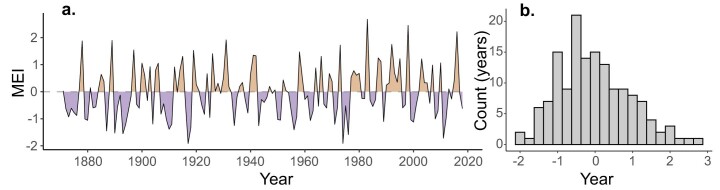
Multivariate ENSO index (MEI) record. Merged MEI v1 and MEI.ext time series used to quantify ENSO strength (a) and the overall distribution of MEI values (b). Positive values represent El Niño conditions, while negative values represent La Niña.

ENSO strength was quantified using the multivariate ENSO index (MEI)^33,34^, which spans the period 1871–2018 (Extended Data Fig. 4). In the USA, the National Oceanic and Atmospheric Administration (NOAA) instead relies on the Niño3.4 index^11^ to officially declare El Niño conditions; however, NOAA also uses MEI values of ± 0.5 as a near-equivalent approximation for moderate El Niño/La Niña years. The MEI captures a broad measure of ENSO strength through sea level pressure and sea surface temperature anomalies in the Pacific Ocean. Other indices exist that target specific regions or facets of the ENSO process but are not considered here.

### Regional ENSO effects

**Fig. 1:**
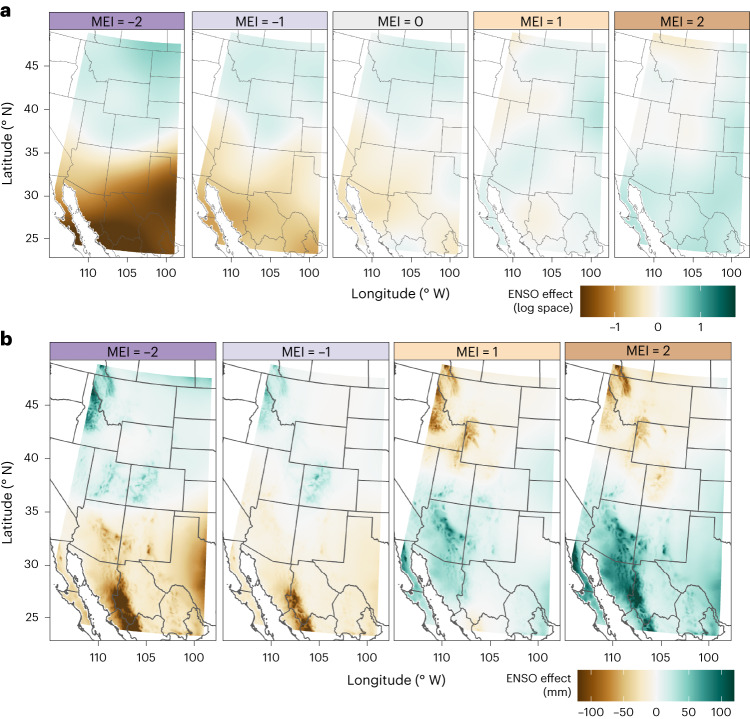
ENSO effects on winter precipitation. **a**, The top row shows the ENSO variable isolated from the first three climatology variables in equation (1). The ENSO effect is presented as anomalies in logarithm space, as in the model (equation (1)). **b**, The bottom row shows the ENSO effect as precipitation anomalies (mm) from neutral ENSO conditions (MEI = 0). Brown colours represent drier than typical, while green represents wetter than typical. Each panel corresponds to an MEI value, ranging from left to right, showing strong La Niña to strong El Niño, with the label colour scheme corresponding to Fig. 3.

On the basis of the full regional model, ENSO teleconnections produce a north–south dipole across western North America, with decreased precipitation in the south during the negative phase (MEI < 0) and increased precipitation during the positive phase (MEI > 0) (Fig. 1a). The opposite is true for the northern pole. The southern centre of precipitation effect appears clearer than the northern in log space (Fig. 1a) due to relatively smaller precipitation values, whereas precipitation differences relative to a neutral year (MEI = 0) indicate similar magnitudes, particularly for high-elevation regions (Fig. 1b and Extended Data Fig. 1). The northern pole is situated north of 40° N, while the southern pole remains south of 35° N, with some northward creep along the eastern edge of the study area, consistent with studies of ENSO in the southeastern USA^17^.

In the southern portion of the dipole, the orographic effect exaggerates changes in absolute precipitation along the windward slope of the Sierra Madre mountains (Fig. 1b). The centre of this effect is located along the Sonora–Chihuahua border (109° W), extending northward into Arizona and New Mexico, particularly during El Niño years. ENSO teleconnections extend eastward along the leeward Mexican Altiplano, indicated by the ENSO term in log space (Fig. 1a), but the absolute magnitude of this precipitation anomaly is far smaller due to a drier overall climatology (Fig. 1b). For our study region, we assume windward corresponds approximately to the western slope, due to the prevailing winter westerlies that predominate across the mid-latitudes where our study area is defined.

For the northern region of the dipole, the ENSO effect is reversed, with increased winter precipitation during La Niña events and decreases during El Niño. The effect is more spatially localized due to a weaker signal and more extreme topography (Fig. 1). Again, topography exaggerates the ENSO effect, producing larger absolute changes for high-elevation stations on the western, windward slopes. The Teton Range, located along the border of Idaho and Wyoming (111° W) is indicative of this pattern and is the focus of the smaller extent northern dipole model.

The regional model explained 62.3% of winter precipitation variance (*r*^2^ = 0.623). The ENSO term proved statistically significant (*P* < 0.05), further supported by increases in the Akaike information criterion (AIC) and Bayesian information criterion (BIC). A purely climatological model without ENSO influence explained 60.3% of variance, suggesting the ENSO term explained an additional 2% of total variance. While seemingly small, this value should be viewed in the context of extreme spatial and elevation precipitation differences across the study area, with median winter precipitation amounts ranging from 4 mm to 700 mm. Climatology therefore rightly represents the majority of variance, but the statistical significance and AIC/BIC indicate that ENSO plays an important interannual role of up to ± 100 mm.

### Secondary constituents

**Fig. 2:**
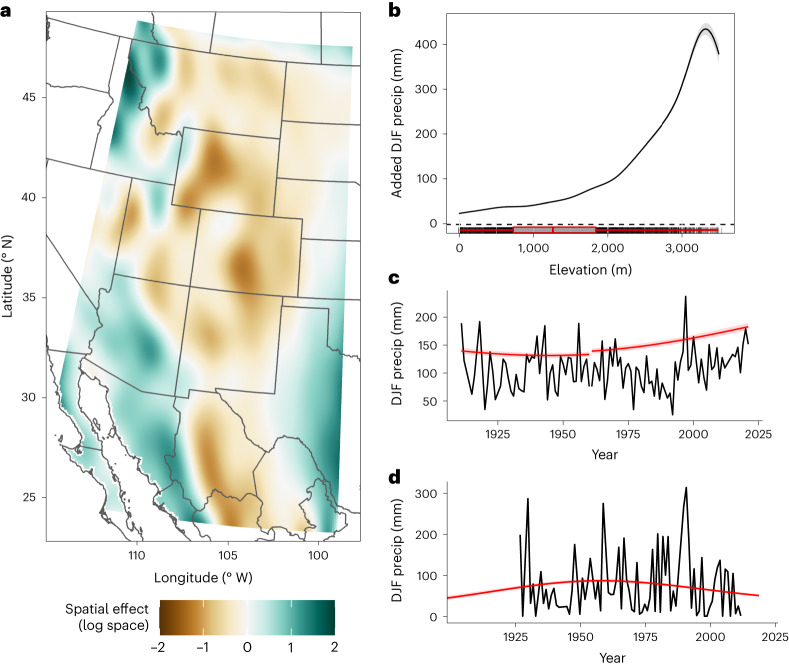
Non-ENSO model terms impacting winter precipitation. **a**–**d**, Isolated non-ENSO model terms from the regional model are shown as anomalies with respect to location only *f*(*x*, *y*) (**a**), elevation only *f*(elev) (**b**) and multi-decadal climate trends *f*((*x*, *y*), year) extracted for sites near the centroid of the North–Wyoming pole (**c**) and the South–Sonora pole (**d**). Panels **a**, **b** and **c**,**d** respectively refer to the first three covariates in equation (1). Together, these terms represent the background climatology, without the effect of ENSO, separated into their constituent parts. The spatial effect (**a**) is shown in original logarithm space for clearer visualization, with positive and negative values adjusting the background climatology. All other panels are plotted in December–February (DJF) precipitation amount (mm), visualizing the orographic increase in precipitation relative to background (**b**) or the change in mean winter precipitation over the twentieth and twenty-first centuries for the north (**c**) and south (**d**). The North–Wyoming climate trend (**c**) uses two gauge locations stitched together because the original gauge was relocated.

Non-ENSO secondary terms, corresponding to the first three terms in equation (1), lend credence to the ability of the regional model to accurately isolate typical orographic effects and reproduce the complex patterns underlying regional precipitation. The spatial term, *f*(lon, lat) representing longitude and latitude, shows an alternating pattern of wet and dry anomalies along the western (windward) and eastern (leeward) slopes, respectively, for each major mountain range (Fig. 2a). This is especially clear where the spatial term bends to follow the Mogollon Rim, which separates lower-elevation Arizona from the high Colorado plateau (35° N). The Mexican Altiplano (108° W) is the most prominent negative region, formed in the eastern rain shadow of the Sierra Madre mountains (Fig. 2a).

The elevation term is nearly linear in log space, producing an exponential increase (Fig. 2b) that doubles winter precipitation for each 0.88 km increase in elevation. This is equivalent to an increase of 120% per km of elevation increase, similar but higher than 75% km^−1^ reported elsewhere for the entire western USA^35^. The resulting precipitation lapse rates therefore increase with elevation: approximately 5, 17 and 70 mm km^−1^ per month for elevation ranges of 0–1,000; 1,000–2,000 and 2,000–3,000 m, respectively (Fig. 2b). These rates are within the range of orographic gradients found elsewhere^10,35,36^, confirming that the model provides a physically realistic basis over which the ENSO signal operates interannually. The reversal above 3,200 m is a result of relatively few extreme elevation gauges and represents a very small proportion of the total site area. This is reflected in higher uncertainty (Fig. 2b). Others have noted dramatically increased variability in the precipitation lapse rate above 3,000 m (ref. ^36^), which may warrant future investigation using higher resolution climate models to explore potential physical causes. Within the context of this study, we believe this reversal is most related to statistical uncertainty, caused by fewer samples covering a very narrow band of high-elevation locations near mountain peaks. The vast majority of the gauges, covering all but the most extreme peaks, follow a physically realistic orographic lapse rate.

Although the effects of topographic enhancement and rain shadowing on precipitation are well understood in general, the isolated spatial and elevation parameters defined here (Fig. 2a,b) represent a secondary benefit of the study, providing the best available regional orographic winter precipitation estimates leveraging the last century and a half of observations, made possible by controlling for climate change and ENSO variability.

Multi-decadal climate trends indicate a century-long precipitation increase in the north (≥40° N), accelerating after 1960, and a decrease in the south (≤32° N) following a mid-century peak (Fig. 2c,d). These patterns are illustrated using two representative locations near the Idaho–Wyoming border (Fig. 2c) and in Quiriego, Sonora, on the western slope of the Sierra Madre range (Fig. 2d). The Sonoran gauge illustrates the importance of the Tweedie distribution (Methods) for incorporating observations near or equal to zero, shown in black. The transition zone (32–40° N) between these two regions shows no consistent long-term winter precipitation trends.

### Dipole models

**Fig. 3:**
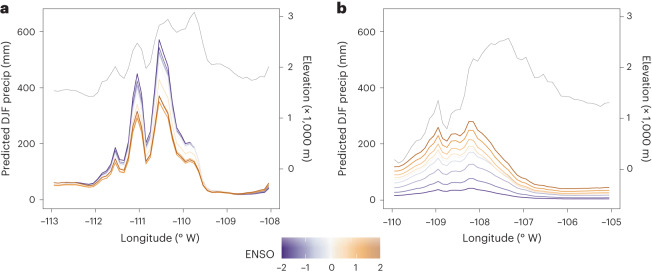
ENSO effects along east–west transects. **a**,**b**, ENSO effect shown across an east–west transect of the North–Wyoming dipole centre (**a**) and the South–Sonora dipole centre (**b**). Colours represent the phase of ENSO, measured by MEI from strong La Niña (MEI = −2) to strong El Niño (MEI = 2). Precipitation is shown on the primary *y* axis. For spatial reference, elevation is plotted in grey and shown on the secondary *y* axis. The *y* axes are identical for both panels to allow relative comparisons.

Independent subset models, referred to as dipole models, were fit to provide a more detailed examination of ENSO effects for the northern and southern dipole centres. Within each dipole model, mountains run approximately north to south, allowing east–west transects to illustrate how elevation interacts with ENSO to modify winter precipitation (Fig. 3). Within their respective model extents, the North–Wyoming model explained 67.1% of winter precipitation, with a root mean square error (RMSE) of 46.9 mm, while the South–Sonora dipole model explained less variance (34.5%), producing a larger RMSE (60.3 mm).

**Fig. 4:**
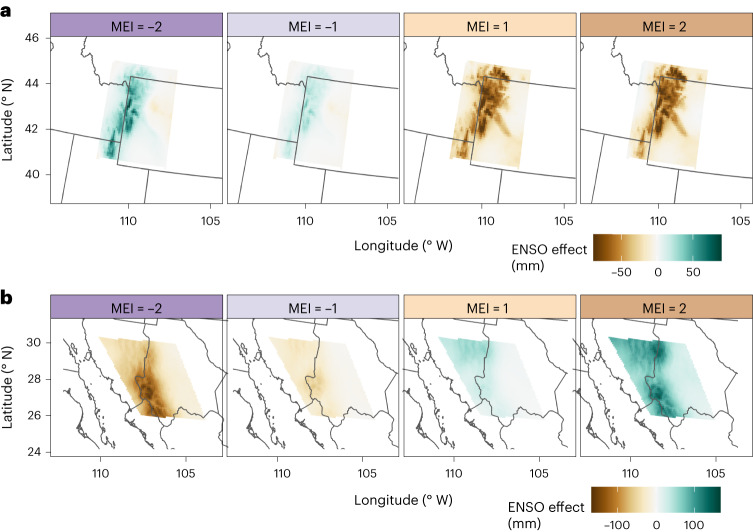
ENSO effects for northern and southern poles. **a**,**b**, Effect of ENSO on winter precipitation, shown as precipitation anomalies from neutral ENSO conditions (MEI = 0), for the North–Wyoming (**a**) and the South–Sonora (**b**) dipole models. Organized similar to Fig. 1, ranging from strong La Niña (left) to strong El Niño (right).

Viewed along the transect bisecting the North–Wyoming model, ENSO impacts on winter precipitation are isolated to the windward slope and increase with elevation, particularly above 2,000 metres (Fig. 3a). Consistent with the broader regional model, winter precipitation increases during La Niña years (MEI < 0) and decreases during El Niño (MEI > 0). East of the mountain peak, interannual differences between ENSO years are negligible (Fig. 3a). These patterns are consistent across the models’ spatial extent (Fig. 4a). The region of El Niño precipitation decrease extends further eastward than the La Niña area of impact, which remains largely isolated on the western slopes (Fig. 4a). Precipitation differences at sites with significant effects typically range from +60 mm to −75 mm for La Niña and El Niño, respectively. The transition between positive and negative phases is discontinuous, meaning there is little gradation with ENSO severity, producing two discrete effects rather than a continuous progression.

The South–Sonora dipole model shows increased winter precipitation during El Niño and decreased precipitation during La Niña (Fig. 3b), in keeping with the regional model (Fig. 1). ENSO teleconnections produce a larger absolute effect for the southern pole than in the north, decreasing winter precipitation to near zero during strong La Niña events and increasing to more than 200 mm during El Niño (Fig. 4). Unlike the North–Wyoming dipole model, the South–Sonora model indicates a more gradual and continuous progression between positive and negative ENSO phases (Fig. 3b). Whereas the largest effect occurs along the western slopes, as expected, the gradual ENSO effect continues east along the leeward slope, probably allowed by a lower topographic peak and more gradual leeward slope (Fig. 3b). A secondary zone of effect near 30° N appears only for precipitation increases during the El Niño phase (Fig. 4b). This spatial anomaly may be due to the northern sites, located near the Chihuahua desert, being near zero precipitation and thus unable to decrease further during La Niña.

### Atmospheric rivers

**Extended Data Fig. 5:**
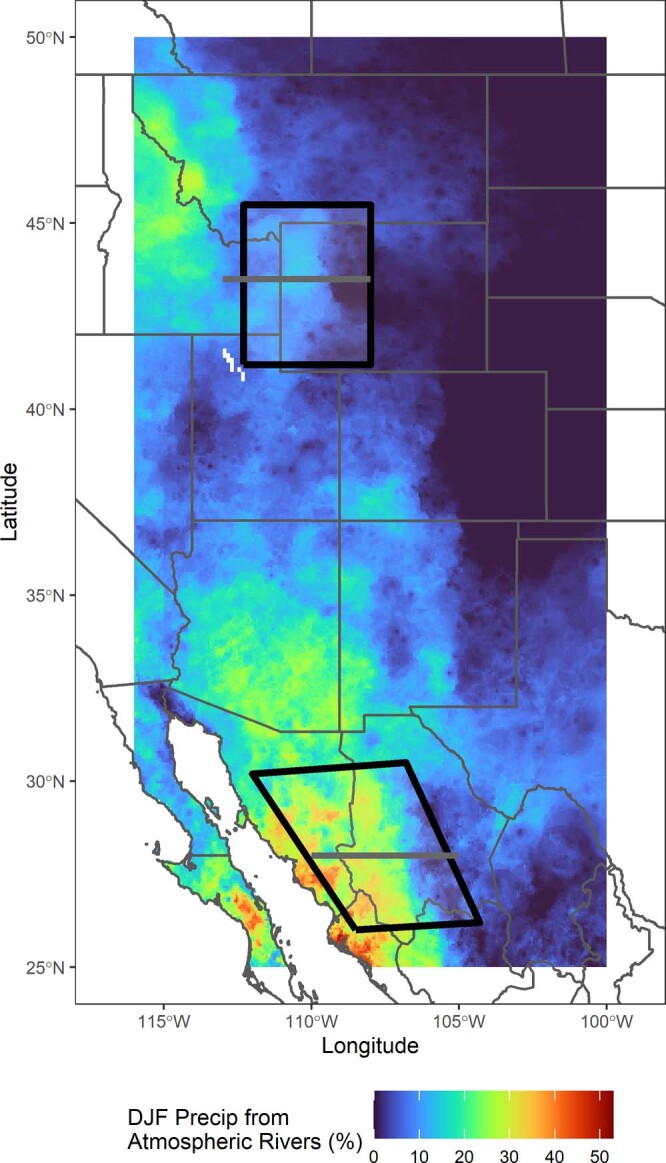
Atmospheric river contributions. Median proportion of DJF precipitation attributable to atmospheric rivers during 1948-2015 using data from^42^.

**Extended Data Fig. 6:**
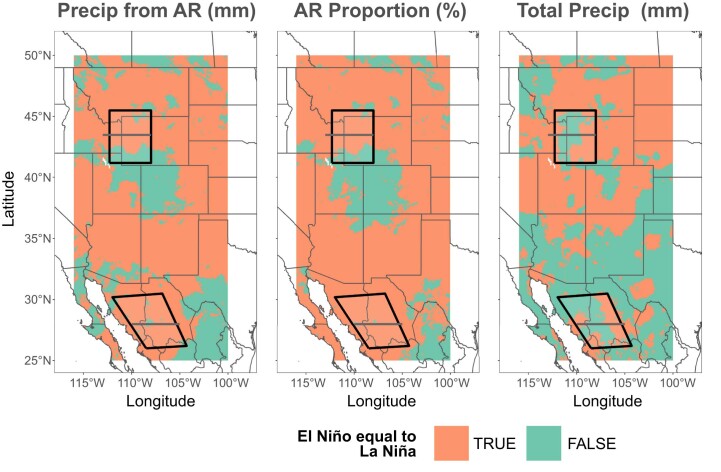
ENSO interaction with atmospheric rivers. Mann-Whitney tests indicating where El Niño and La Niña conditions are significantly different, where years are categorized based on NOAA’s threshold of MEI = ± 0.5. Years between -0.5 and 0.5 are considered neutral and not used for this comparison test. The compared variables are: DJF precipitation associated with atmospheric rivers (left), proportion of DJF precipitation associated with atmospheric rivers (center), and total DJF precipitation (right). Green regions show significantly different behaviors.

**Extended Data Fig. 7:**
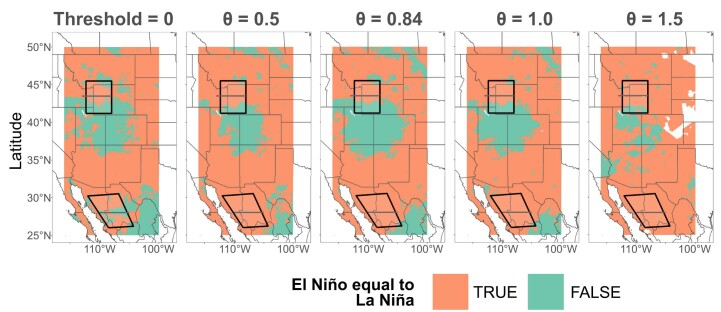
Sensitivity test for atmospheric river contribution. Similar to Extended Data Figure 6. Results of Mann-Whitney test showing where El Niño and La Niña years exhibit statistically significant differences in the proportion of DJF precipitation associated with atmospheric rivers. Each figure uses a progressively high threshold to distinguish between neutral conditions and El Niño or La Niña years. Green regions show significantly different behaviors. Significant regions remain consistent until a high threshold decreases the number of available years in the dataset.

Prior studies indicate that El Niño events increase atmospheric river landfalls in coastal North America, particularly in the Pacific Northwest^18,37–39^. The equivalent and opposite effect on atmospheric rivers during La Niña years has been more difficult to identify^37,38^. Atmospheric rivers are far more important for winter precipitation along the Pacific coast (45% to 60% of winter precipitation) than in the Intermountain West, where the median atmospheric river contribution to DJF precipitation is nearer to 0–15% due to blocking effects from California’s Sierra Nevada mountains^40–42^ (Extended Data Fig. 5). Two exceptions to this blocking are the extreme northwest and southernmost portions of our study area, where atmospheric rivers contribute 20–30% and 20–40% of winter precipitation, respectively (Extended Data Fig. 5). To determine whether atmospheric river disruptions can explain the ENSO effects identified here, we compared atmospheric river contributions^42^ during El Niño/La Niña winters. While El Niño and La Niña significantly affected the amount and proportion of atmospheric river contribution in some parts of the Intermountain West, these tend to be low-lying areas least subject to Sierra Nevada blocking (Extended Data Fig. 6). However, the regions with greatest ENSO effects identified by the regional and dipole models (Figs. 1 and 4) are not significantly driven by changes in atmospheric river behaviour. These findings were stable, regardless of the threshold used to define El Niño and La Niña phases (Extended Data Fig. 7).

It should be noted that most of the underlying precipitation stations used to generate the atmospheric river precipitation estimates^42,43^ are identical to those used in our models. However, our models use raw station data, without spatial interpolation to create gridded estimates. This allows for an additional 80 years of data in some locations, doubling the record length for ENSO comparisons in our study. In testing, our modelled orographic lapse rates (Fig. 2b) were similar to those used in the pre-processing step described previously^43^.

### El Niño outliers

**Extended Data Fig. 8:**
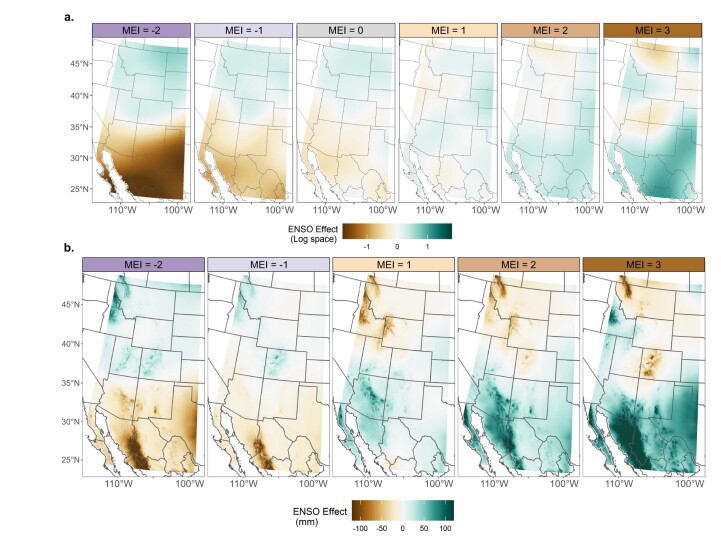
Regional ENSO effects including outliers. Similar to Fig. 1, but expanded to include MEI = +3. **a**, The top row shows the ENSO variable isolated from the first three climatology variables in equation (1). The ENSO effect is presented as anomalies in logarithm space, as in the model (equation (1)). **b**, The bottom row shows the ENSO effect as precipitation anomalies (mm) from neutral ENSO conditions (MEI = 0). Brown colours represent drier than typical, while green represents wetter than typical.

**Extended Data Fig. 9:**
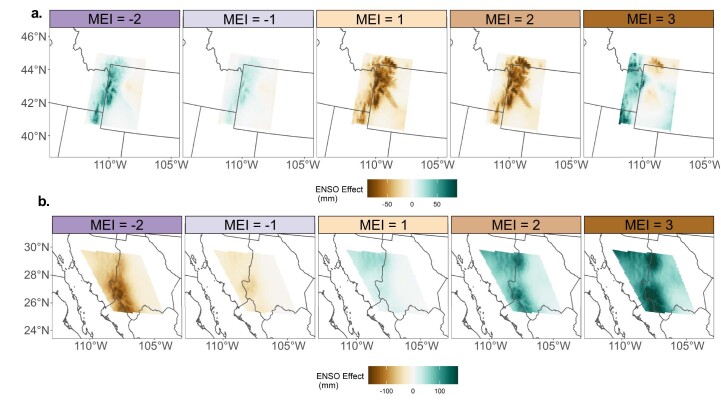
ENSO effects for northern and southern poles including outliers. Similar to Fig. 4, but expanded to include MEI = +3. **a**,**b**, Effect of ENSO on winter precipitation, shown as precipitation anomalies from neutral ENSO conditions (MEI = 0), for the North–Wyoming (**a**) and the South–Sonora (**b**) dipole models.

The available historical record contained three extreme positive outliers (MEI > 2) but no equivalent negative outliers (MEI < −2) (Extended Data Fig. 4). The most extreme positive year, 1983 (MEI = 2.68), produced wet anomalies in the Snake River Valley and Teton Range, counter to the prevailing drier ENSO effect. This did not occur during the two less extreme outlier years (1998, 2016). In the model, the unique 1983 event acted as a high leverage outlier, greatly increasing uncertainty for extreme positive El Niño years. When extrapolated well beyond the strongest El Niño on record (MEI = 3), the model predicts a slight reversal of the established ENSO effects for this region with extremely large uncertainty bounds. The large uncertainty and outsized effect of the single 1983 outliers suggests this is a modelling anomaly due to a paucity of other extreme years, which may also explain why others have found uniquely low correlations in the region^19^. Ultimately, we chose to retain 1983 in the model,but only present ENSO effects for MEI < 2, encompassing all but the most extreme 2% of recorded events. For completeness, we present extrapolated effects in Extended Data Figs. 8 and 9.

Further evidence for 1983 acting as an atypical outlier comes from understanding its record-breaking atmospheric conditions. During the winter of 1983, a strong ridge of high pressure formed over Alaska, coinciding with an amplification of the jet stream during December. The resulting atmospheric blocking caused a polar vortex with record-breaking cold temperatures and high pressure across much of the central USA^44,45^. The western edge of the persistent blocking pattern was located near the Teton mountains, in turn creating a low pressure trough over the Snake River Valley to its west that generated record-breaking high single-month snow totals, in stark contrast to the surrounding cold and dry region. This extreme behaviour may have its origin in the progression of the 1983 El Niño that developed in the central Pacific and propagated eastward^34,46–48^. Such central Pacific Modoki events^49^ have been shown to affect moisture transport differently than canonical ‘eastern Pacific’ El Niño events^39^ and the MEI used here was not designed to distinguish between these different ENSO ‘flavours’^34^.

## Discussion

Our objective here was to disentangle orographic effects and climate trends from ENSO teleconnection impacts on the Intermountain West’s winter precipitation using ground-based observations with long records. Our findings build upon prior studies that identify a dipole effect on winter precipitation across the Intermountain region due to ENSO, with northern increases during La Niña and southern increases during El Niño^13,15,16,50,51^. However, we additionally offer several novel insights due to explicitly modelling the interplay between ENSO teleconnections and orographic effects using a new approach. Once the complicating effects of elevation and climate change are accounted for, a clearer measure of ENSO impacts emerges, particularly for the northern half of the dipole, which has historically proven more elusive^19,30^.

We find that orographic effects amplify the ENSO signal by up to ± 100 mm, primarily for high-elevation sites along western slopes. Along representative transects, this represents an approximately 5.8 and 2.3 times increase in the range of ENSO-induced precipitation changes for the northern and southern poles, respectively, when comparing highest elevations to windward foothill locations. By using irregularly spaced gauges, this effect is clearer than in previous studies that rely on regional averages^16,30^ or percent change^17,19^. Regional averages preclude detailed orographic analyses, while percent change analyses can distort effects in semi-arid regions with negligible or zero precipitation years. Use of a GAM model here permits direct use of irregularly spaced instrumental records over nearly a century and a half (1871–2018; Extended Data Figs. 2 and 3), which in turn provides an unprecedented ability to isolate ENSO signals from orographic effects and multi-decadal climate trends.

The more stable and clearer view of ENSO effects has also brought into sharp focus several new behaviours. First, we detected a notable difference in spatial extent between the northern and southern dipole centres. Where the ENSO signal in the northern dipole dissipates abruptly along its steep eastern slope, the ENSO signal for the southern pole extends across the Mexican Altiplano, probably due to more gradual slopes. Second, we detected, for the first time, a latitudinal difference between the positive and negative phases for each dipole centre. In the north, the precipitation increase during La Niña has a more southern zone of impact, whereas the positive El Niño phase is centred further north. Latitudinal differences are even more pronounced for the southern dipole, due to climatology that permits El Niño increases but limits decreases during La Niña because of a minimum precipitation bound at zero.

Another notable finding was the gradual and consistent effect of ENSO on winter precipitation for the southern pole, contrasted with more sudden and discrete changes in the northern region of the precipitation dipole. For water managers seeking to operationalize this finding, it suggests that forecasts for the northern dipole centre could focus primarily on ENSO phase, whereas more quantitative estimates are needed for southern regions. We hypothesize this is why the southern pole is more readily detected in prior studies that often rely on linear models, contrasted with nonlinear approaches used here.

We also showed increasing winter precipitation trends in the north and decreasing trends in the south, particularly during the latter part of the twentieth century. This broadly agrees with prior trend studies^24–26^, but our new approach is capable of capturing nonlinear patterns, such as an ongoing acceleration of northern precipitation increases and a mid-twentieth century lull and reversal in the south. Though statistically significant, precipitation trends remained smaller in magnitude ( < 40 mm) than ENSO-driven variability ( ≈ 50–75 mm) and the stochastic component of interannual variability ( ≈ 50–100 mm).

The orographic enhancements shown here are caused by the interaction of traditionally understood orographic mechanisms^10^ with predominant atmospheric circulation patterns, driven by ENSO drivers and also subject to internal atmospheric variability^13,14^. Orographic precipitation effects are caused when moist air masses are advected by lower tropospheric winds towards mountainous barriers where they are lifted vertically, increasing precipitation on the windward side^10^. These effects are typically proportionate to wind speed. In the Intermountain West, the North American Cordillera and its subranges act as these barriers^30,52^. The near-exponential orographic precipitation increase along windward slopes (Fig. 2b) exaggerates the diffuse effect of ENSO (Fig. 3); that is, El Niño (La Niña) events result in increased westerly flow in the southern (northern) pole, resulting in a proportionate increase in orographic forcing of precipitation. Accordingly, an increasingly wide gap occurs between precipitation anomalies between El Niño and La Niña years with increasing elevation. This effect is clearest when comparing absolute precipitation, as done here.

As described previously, El Niño conditions in the Pacific Ocean cause a wave train that typically extends the Pacific jet towards the southwestern coast of the USA and northern Mexico, increasing precipitation and atmospheric river frequency in the southern pole of our model^12,18,53^. The northern pole is subject to greater mechanistic complexity, being located towards the continent’s interior and east of California’s Sierra Nevada range that captures or deflects much of the direct on-shore moisture^41^. Atmospheric rivers play a smaller and largely not statistically significant role in ENSO effects for the northern pole centre (Extended Data Fig. 6). Instead, during La Niña years, conditions exist for the Pacific jet to deflect poleward along the US–Canadian border, bringing moisture into the northern Intermountain West where overall precipitation increases and topography exaggerate these increases for high-elevation, windward sites^13,15,20,30^. Recent studies have suggested that this La Niña mechanism probably interacts with the Pacific North American Pattern, which is correlated with ENSO, or the spatially similar Atlantic Quadpole Mode^14,15,20,41^. These patterns modify the east–west location of a high pressure centre along the US–Canadian border, either permitting intrusion of ENSO-driven moisture to the northern Intermountain West or blocking it along the Pacific Coast. Further research should explore the moderating effects of these North American patterns or alternative measures of ENSO^13,39^.

The Intermountain region of western North America is consistently under water stress^1^ and heavily depends on winter precipitation for irrigation and municipal water demands. The topography of this region offers a challenge for disaggregating ENSO effects from orographic and long-term climate effects, but the GAM approach developed here provides an increasingly accurate model to predict precipitation in this topographically challenging environment. Furthermore, its use could be expanded to better understand local orographic modification of teleconnection effects on precipitation in other montane regions globally. An additional important feature of the GAM model is that it depends only on winter MEI as a predictor variable. If combined with seasonal ENSO forecasts^13,31^, the fitted model would produce high spatial resolution winter precipitation forecasts in units of depth with usable confidence intervals for use by water planners to anticipate spring reservoir release decisions or to inform irrigators of forecast shortfalls.

## Methods

This study uses generalized additive models (GAMs), a nonlinear form of regression, to model winter precipitation across the Intermountain West of North America using predictor covariates to account for location, elevation, multi-decadal climate trends and interannual effects from ENSO, as measured by the MEI^33,34^. Once a model is fitted, the effects of these covariates may be quantified separately or together to better explore their impacts. The specific modelling approach was separated into two experiments based on the model domain, referred to as the regional and dipole models, respectively. The regional model captures the Intermountain West of North America extending to cover both the north and south dipoles of the ENSO effect. The dipole model uses separate models for the northern and southern dipole centres. Both models use a similar format, permitting analyses at different scales and cross-model verification regarding the stability of findings.

### GAMs

GAMs use nonlinear spline functions and higher-dimensional spline-based surfaces as additive predictors to build a nonlinear multiple regression model^54–56^. The flexibility of GAM splines are controlled by the knots, which act as control points, and a penalty function that counteracts overfitting due to excess ‘wiggliness’ in the splines^57^. The number of knots was selected based on repeated fitting using the AIC, while the penalty was selected based on generalized cross validation (GCV)^57,58^. Specific details are provided for the regional and dipole models in the following subsections.

For all models, the variable of interest was mean precipitation for December to February, *P*_DJF_. Because *P*_DJF_ precipitation tends to be positively skewed with potential for zero values, the model utilized a Tweedie distribution^59,60^ for the dependent variable. The Tweedie distribution is an exponential dispersion model, which can approximate the Poisson or gamma distributions while simultaneously allowing for zeros^59,60^. This distribution uses three parameters: mean, dispersion and power (*μ*, *ϕ* and *ρ*, respectively). Values of *ρ* were limited to the range between 1 and 2, which allows for a point mass at zero precipitation and a skewed right distribution for detectable values, simulating typical distribution choices for seasonal precipitation^61^. Variance around the estimate for a Tweedie distribution is therefore:

2 $$\mathrm{Var}({P}_\mathrm{DJF})=\phi {\mu }^{\rho }$$

and the point mass for zero precipitation is:

3 $$f\,(0;\mu ,\phi )=\mathrm{exp}\left(-\frac{{\mu }^{(2-\rho )}}{\phi (2-\rho )}\right)$$

where all parameters are as described above. The GAM models in this study were fit using the ‘-bam()’ function from the mgcv package^62,63^ in R. The ‘bam()’ function is used for GAM models with large datasets and fits an initial data subset before fitting the full model to limit the parameter search space and decrease memory requirements^62^.

### Regional model

The regional model study area was defined by a rectangle from −25° to 50° N and −116° to −99° E (Extended Data Fig. 1). This choice was made to provide enough north–south extent to cover both centres of assumed ENSO influence in the Intermountain West. The east–west extent was chosen primarily to isolate the Rocky Mountains through the centre of northern ENSO influence, while also limiting spatial interpolation across the Pacific Ocean. The regional model for winter precipitation is:

4 $$\log\Big({P}_{{{{{\rm{DJF}}}}}_{i,\,j}}\Big)=f{(x,y)}_{j}+f{({{{\rm{elev}}}})}_{j}+f\Big({(x,y)}_{j},{{{{\rm{year}}}}}_{i}\Big)+f\Big({(x,y)}_{j},{{{{\rm{MEI}}}}}_{i}\Big)$$

where the target variable, ${P}_{{{{{\rm{DJF}}}}}_{i,\,j}}$, represents winter precipitation for a given year, *i*, at a precipitation gauge, *j*. A log transformation was applied to the estimate as is common to ensure strictly positive estimates for the Tweedie distribution. The two other Tweedie parameters, *ϕ* and *ρ*, were estimated simultaneously and held constant across the model.

As described previously, each model seeks to explain winter precipitation as the sum of nonlinear predictors representing location, elevation, climate trends and the interannual effect of ENSO. The first model predictor *f*(*x*, *y*) captures the effect of gauge location using a tensor product spline^56,57^. A tensor product spline is simply a multidimensional spline surface that can allow different smoothness and units for either direction. In this case we can model the portion of the response attributed to a gauge’s location using a tensor product spline surface with dimensions for the spatial coordinates x and y, with coordinates measured in metres using the USA Contiguous Albers Equal Area Conic projection. The second predictor, *f*(elev), models the additional effect of elevation. These two terms do not change temporally. So, when summed, they model the mean climatology for any location.

The third predictor, *f*((*x*, *y*)_*j*_, year) captures climate trends, modelled spatially across the region. This is accomplished by another tensor product spline with an added temporal dimension, year, that permits slow changes in mean precipitation through time. To ensure this term captures only long climate trends, the flexibility of the spline term was designed with control points (knots) ever 30 years to mimic the 30-year reference period recommended by the World Meteorological Organization for measuring climate baselines^64^. Once added to the first two predictors, the resultant model would estimate typical winter precipitation at any location through the centuries.

The final predictor, *f*((*x*, *y*)_*j*_, MEI_*i*_), captures the effects of ENSO, modelled spatially. This predictor is the key component for identifying the effect of ENSO on winter precipitation. In this case, ENSO is measured via the MEI index for each year.

For model validation, the regional model was compared against a non-ENSO null model, fitted without the final ENSO term and designed to represent mean conditions. These models were compared based on the AIC and the BIC to evaluate goodness of fit and whether inclusion of the ENSO term provides a significant model improvement. Further, parameter estimates between the regional model and this non-ENSO null model were compared to ensure that the other predictor effects remained consistent after inclusion of the ENSO predictor.

### Dipole model

Separate dipole models were fit for the northern and southern poles. The northern Dipole model, referred to as the North–Wyoming model, was bounded by 41.2° to 45.5° N and −112.3° to −108° E (Extended Data Fig. 1). This region was chosen to highlight an important centre of ENSO effect with large elevation relief but also to avoid the Uinta mountain range, which runs east–west. This east–west mountain orientation is uncommon in the North American Cordillera, so was purposefully removed. The southern dipole model, referred to as the South–Sonora model was drawn as a parallelogram to mimic the area of ENSO impacts along the Sierra Madre Occidental mountains. This region (Extended Data Fig. 1) is bounded by 26° N to 30.5° N and −112° E to −104.3° E.

The dipole model is nearly identical to the regional model, with two minor modifications:

5 $$\log\Big({P}_{{{{{\rm{DJF}}}}}_{i,\,j}}\Big)=f{(x,y)}_{j}+f{({{{\rm{elev}}}})}_{j}+f({{{{\rm{year}}}}}_{i})+f({{{{\rm{MEI}}}}}_{i})+f\Big({(x,y)}_{j},{{{{\rm{MEI}}}}}_{i}\Big)$$

The first modification is that the multi-decadal climate trend, *f*(year), was simplified to remove the spatial component. The assumption inherent in this model choice is that anthropogenic climate change or natural climate variability produce trends of a similar direction across such a limited region, though with different absolute magnitudes once climatology and orographic effects are included. The second modification is the separation of the ENSO effect into an average effect, *f*(MEI_*i*_), and a spatially distributed effect, *f*((*x*, *y*)_*j*_, ENSO_*i*_). The average effect was not feasible in the regional model because the northern and southern locations had opposed ENSO responses. Ultimately, this latter model choice made little difference, creating a nearly identical response surface as initial testing with a single MEI predictor.

### Precipitation gauge data

Winter precipitation was based on the Global Historical Climatology Network (GHCN-D) daily dataset, a database of instrumental climate observations from land surface stations that has been subjected to quality assurance reviews^65^. Only gauges within the bounding box were considered (Extended Data Fig. 1). GHCN-D precipitation data were then processed to extract the December–February mean precipitation for each year. The dataset was then filtered to include only those years with at least 82 days of recorded precipitation (up to seven days missing out of three months) and further filtered to include only those gauges with at least 20 years of complete *P*_DJF_ data.

This filtering process produced 4,287 unique precipitation gauges spanning a period from 1849 (Santa Fe, NM) to 2021. There is a steady increase in gauge availability from 1871 to 1950, followed by a rapid increase in the 1950s and relatively consistent availability until the present (Extended Data Fig. 2). No gauges were available before 1900 in Mexico but began soon thereafter (Extended Data Fig. 3). For calibration purposes, each time series was limited to the ENSO record, 1871 to 2018. Elevation for the resulting gauges ranges from 0 to 3,536 metres, with a median of 1,333 metres and mean of 1,389 metres. The elevation distribution is skewed right (Extended Data Fig. 1b) with fewer high-elevation gauges. For gridded predictions among the gauge locations, 90-m gridded elevation data from the Shuttle Radar Topography Mission (SRTM) were used^66^.

### ENSO data

Historical estimates of the ENSO were based on the MEI. The MEI in turn is based on a principal component analysis of six observed variables over the tropical Pacific: sea level pressure, surface zonal and meridional wind, sea surface temperature, surface air temperature and cloudiness^33^. For the purposes of this study, we combine the MEI v1 with the extended MEI (MEI.ext)^34^ to obtain an extended MEI record that continues towards the near present. Where the MEI v1 covers the period 1950–2018, the MEI.ext (1871–2005) more than doubles this record length, while retaining strong agreement during the common period. The MEI.ext is based on similar principles but uses a simplified definition with fewer variables: Hadley Centre reconstructed sea surface temperature and sea level pressure fields (HadSST2 and HadSLP2).

The MEI.ext and MEI v1 were merged, with the MEI v1 taking precedence for years when they overlap. Mean absolute error between the two series for the winter period Dec–Jan is 0.18 with the MEI v1 exhibiting a slight negative mean bias (−0.083), meaning that MEI v1 values are consistently lower than the equivalent MEI.ext. This is exceptionally small relative to the typical range of the MEI between −2 and 2. Given this strong agreement, we felt confident in combining the records, with preference given to the MEI v1 because of its larger instrumental basis. The MEI has strong agreement with other ENSO measures. For example, the pairwise correlation between the HadISST Niño 3.4^67^ SST index and the MEI v1 and MEI.ext during the cool season, November–February (NDJF) are 0.964 and 0.970, respectively. The boreal winter, used in this study, was found to have the best correlation between MEI.ext and four common ENSO indices^34^.

The MEI is calculated for 12 sliding bimonthly seasons. For this study, the December–January ENSO index was used to represent annual winter ENSO because it was the period of highest correlation with *P*_DJF_ across the study region. MEI values approximate a normal distribution (mean = 0, standard deviation = 1), with some positive outliers. This slight asymmetry towards positive outliers has been noted previously as a consequence of ENSO sea surface temperature processes^68^ and was noted in the development of the MEI index^34^. The three positive outliers occurred in 1983 (2.68), 1998 (2.46) and 2016 (2.22). ENSO variance underwent a lull in the middle portion of the record (1940s to 1970s)^34,69^.

### Atmospheric rivers

The extent to which ENSO-driven precipitation anomalies can be attributed to atmospheric river changes was evaluated using the Scripps Institution of Oceanography (SIO)-generated catalogue of atmospheric rivers (SIO-R1) in western North America between 1948 and 2017^42^. This catalogue uses National Centers for Environmental Prediction–National Center for Atmospheric Research (NCEP–NCAR) reanalysis data^70^ to define atmospheric rivers making North American landfall wherever daily mean integrated vapour transport was greater than 250 kg m^−1^ s^−1^ for at least two consecutive days. The spatial footprint of atmospheric rivers was then used to categorize gridded precipitation estimates interpolated from land-based stations^43^ as either originating from an atmospheric river or not^42^. This process takes the coarser atmospheric reanalysis and produces a finer 6 × 6-km spatially resolved precipitation (Extended Data Fig. 5).

To test the effect of atmospheric rivers on DJF precipitation, we performed a Mann–Whitney *U*-test^71,72^, a non-parametric test for significant differences between the median of El Niño and La Niña years in three separate tests comparing: (1) the amount of atmospheric river precipitation (mm), (2) the amount of total precipitation (mm) and (3) the proportion of atmospheric river precipitation (% of total) (Extended Data Fig. 6). These tests were performed at each grid cell using a two tailed test (*α* = 0.05) that assumes an alternative hypothesis of med[*P*(MEI > *θ*)] ≠ med[*P*(MEI < − *θ*)], where med[] is the median of winter precipitation, *P*, for El Niño and La Niña years, defined as MEI being above or below the threshold *θ*. To test the sensitivity of results to the threshold, we considered 5 thresholds: 0, 0.5, 0.816, 1 and 1.5 (Extended Data Fig. 7). These represent common ENSO definitions ranging from mild to severe.

### Reporting summary

Further information on research design is available in the Nature Portfolio Reporting Summary linked to this article.

### Supplementary information

Reporting Summary

## Data Availability

All data and digital output are available via an open access Zenodo repository^63^.

## Code availability

All code, data and instructions to reproduce the results are available via an open access Zenodo repository^63^. This code has been tested to generate all figures and tables presented in this paper.
